# A novel efficient eggplant disease detection method with multi-scale learning and edge feature enhancement

**DOI:** 10.3389/fpls.2025.1666955

**Published:** 2025-09-18

**Authors:** Hao Sun, Rui Fu, Dae-Ki Kang

**Affiliations:** ^1^ Shandong Facility Horticulture Bioengineering Research Center, Weifang University of Science and Technology, Weifang, China; ^2^ Department of Computer Engineering, Dongseo University, Busan, Republic of Korea

**Keywords:** eggplant disease detection, deep learning, edge feature enhancement, multi-scale learning, object detection

## Abstract

In the context of the rapid development of smart agriculture, the detection of crop diseases remains a critical and challenging task. The diversity in eggplant disease scales, disease edge features, and the complexity of planting backgrounds significantly impact disease detection effectiveness. To address these challenges, we propose an eggplant disease detection network with edge feature enhancement based on multi-scale learning. The overall network adopts a “backbone–neck–head” architecture: the backbone extracts features, the neck performs feature fusion, and a three-scale detection head produces the final predictions. First, we designed the Multi-scale Edge Information Enhance (CSP-MSEIE) module to extract features from different disease scales and highlight edge information to obtain richer target features. Second, the Multi-source Interaction Module (MSIM) and Dynamic Interpolation Interaction Module (DIIM) sub-modules were designed further to enhance the model’s capacity for multi-scale feature representation. By leveraging dynamic interpolation and feature fusion strategies, these sub-modules significantly improved the model’s ability to detect targets in complex backgrounds. Then, leveraging these sub-modules, we designed the Multi-scale Context Reconstruction Pyramid Network (MCRPN) to facilitate spatial feature reconstruction and hierarchical context extraction. This framework efficiently combines feature information across multiple levels, strengthening the model’s ability to capture and utilize contextual details. Finally, we validated the effectiveness of the proposed model on two disease datasets. It is noteworthy that on the eggplant disease data, the proposed disease detection model achieved improvements of 4.7% and 7.2% in mAP50 and mAP50–95 metrics, respectively, and the model’s frames per second (FPS) reached 270.5. This detection network provides an effective solution for the efficient detection of crop diseases.

## 1 Introduction

**Figure 1:**
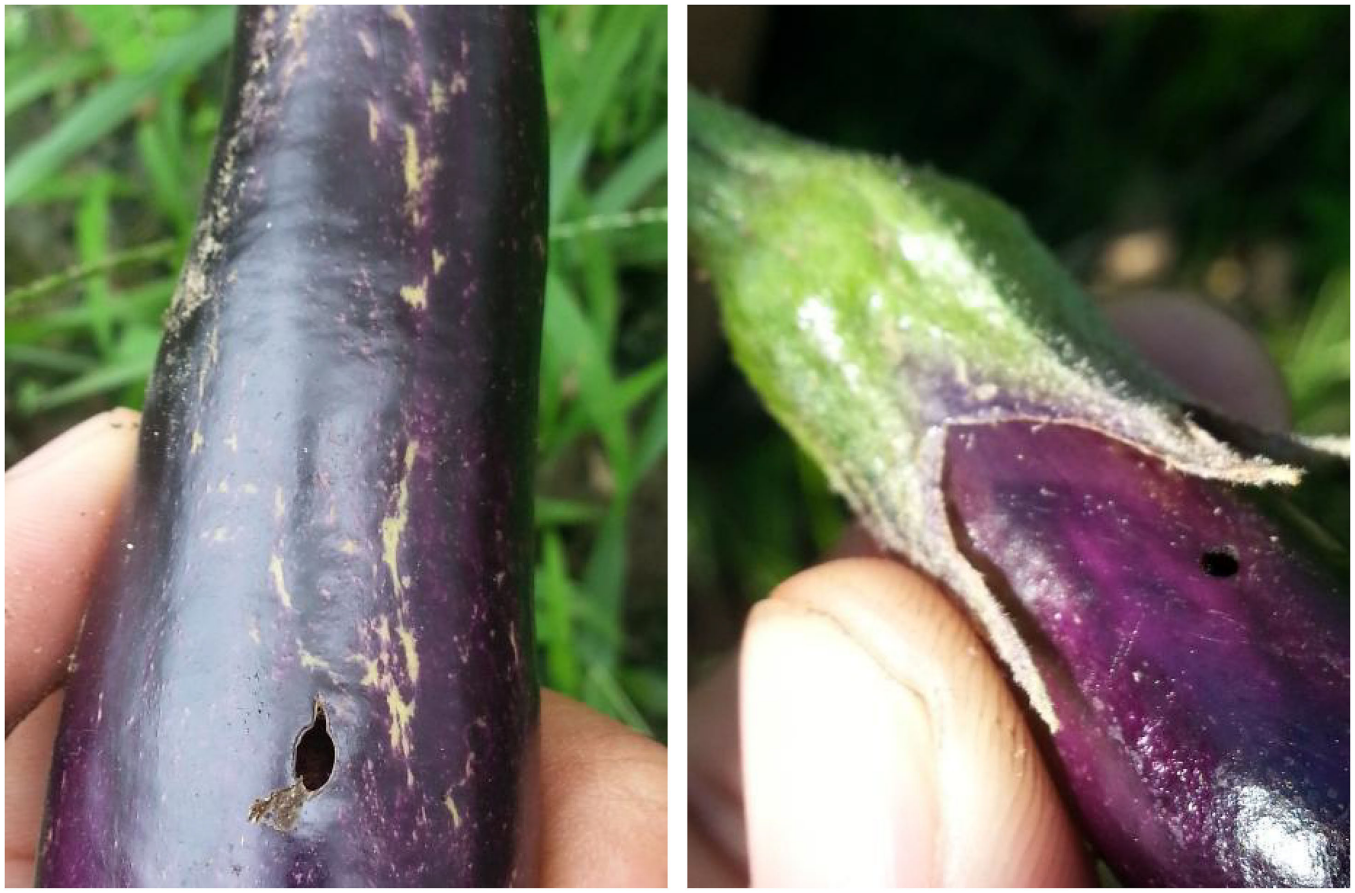
Status of eggplant cultivation, highlighting that very small, concealed fruit diseases (e.g., Fruit Borer) are often missed during field inspection.

Eggplants are widely cultivated and highly valued for their rich content of dietary fiber and essential vitamins. They play a crucial role in improving global dietary patterns and promoting nutritional balance. Advancements in agricultural technology and the rapid growth of international trade have significantly increased both the cultivation area and total production of eggplants in recent years. By 2022, global eggplant production had exceeded 59 million tons, and the cultivation area surpassed 1.89 million hectares, underscoring its importance in modern agriculture (Yan et al., 2024). However, the expansion of cultivation has made eggplants more vulnerable to diseases and pest infestations, as shown in **Figure 1**, especially under increasingly complex and unpredictable climate conditions. Common problems such as yellow spot disease, fruit rot, and pest infestations severely threaten eggplant yield and quality, resulting in significant economic losses for growers (Liu and Wang, 2021).

Efficient disease management remains a central challenge in agricultural production. Traditional management methods mainly rely on manual inspection and chemical control, both of which have notable limitations. Manual inspection is time-consuming and prone to errors due to subjective judgment and reliance on individual experience, making it unsuitable for large-scale modern agriculture. Moreover, the growing reliance on pesticides to combat frequent disease outbreaks not only raises production costs but also increases pathogen resistance, posing additional threats to the environment and food safety. Therefore, developing accurate and efficient disease detection and management technologies is imperative.

In recent years, automated detection technologies have advanced rapidly in agriculture, offering innovative solutions for addressing eggplant diseases. For example, spectral analysis has been preliminarily applied to eggplant disease identification. However, the complexity of processing high-dimensional spectral data and the associated information loss during dimensionality reduction have become major development bottlenecks (Wu, 2018). Additionally, texture-based feature extraction algorithms combined with classification models have shown moderate effectiveness in eggplant disease classification (Xie and He, 2016). However, these methods depend on manual feature extraction, suffer from pixel-level information loss, and exhibit high computational complexity, limiting their scalability and practical use. The emergence of machine learning has introduced promising solutions for crop disease recognition. Convolutional Neural Network (CNN)-based models have been successfully applied to eggplant disease recognition, demonstrating notable advantages over traditional approaches. However, early machine learning models mainly focused on classification tasks (Krishnaswamy Rangarajan and Purushothaman, 2020; Maggay, 2025; Theckedath and Sedamkar, 2025), neglecting the critical aspect of disease localization. This limitation prevents these models from fully replacing manual inspection, as accurate localization is essential for targeted treatment and intervention.

The rise of computer deep learning technology has driven object detection models toward greater efficiency and precision. These models can classify diseases and accurately localize them in images, representing a breakthrough in automated crop disease detection. Object detection models are typically categorized into two types: single-stage and two-stage detectors. Representative two-stage models include SSD (Tian et al., 2023), Faster R-CNN (Ren et al., 2017), and RetinaNet (Math and Dharwadkar, 2023). These models first generate region proposals or candidate bounding boxes, followed by classification and fine-grained localization within those regions. In contrast, single-stage models such as the YOLO (You Only Look Once) (Redmon et al., 2016; Redmon and Farhadi, 2017; Bochkovskiy et al., 2020; Redmon and Farhadi, 2018; Jocher et al., 2022; Wang et al., 2022; Redmon and Farhadi, 2025; Wang et al., 2024b; Li et al., 2022) series are widely recognized for their real-time performance and high accuracy. Recent research has increasingly focused on enhancing YOLO models for crop disease detection. For example, Liu et al. proposed a YOLOv5 variant with a novel loss function to detect tomato brown rot (Liu et al., 2023). Wang et al. integrated a Transformer into YOLOv8 to enhance tomato disease detection, significantly improving its ability to capture detailed disease features (Wang and Liu, 2024). Jiang et al. combined the Swin Transformer with CNN to optimize YOLOv8’s feature extraction, improving detection performance for cabbage diseases under complex conditions (Jiang et al., 2024). Liu et al. introduced a multi-source information fusion approach based on YOLOv8 to enhance detection accuracy across multiple vegetable diseases (Liu and Wang, 2024). Moreover, some researchers have further improved detection performance on target images by employing edge-image enhancement (Wang et al., 2023) and additional image-preprocessing techniques (Wang et al., 2024c).

Although these enhanced YOLO models have shown progress, they primarily focus on leaf diseases, small datasets, and parameter tuning. However, the impact of scale variations in fruit disease regions and edge features under complex backgrounds on detection accuracy remains underexplored. To address this critical gap in current eggplant fruit disease detection methods, we propose an eggplant disease detection network with edge feature enhancement based on multi-scale learning. The key contributions of this paper are outlined as follows:

- We develop the Multi-scale Edge Information Enhancement (CSP-MSEIE) module, which extracts features across multiple disease scales and highlights the edge characteristics of affected regions, enabling richer and more comprehensive target representations.
- We develop the Multi-source Interaction Module (MSIM), Dynamic Interpolation Interaction Module (DIIM), and Multi-scale Context Extraction Module (MCEM), which enhance the model’s capacity to capture multi-scale features and improve target detection accuracy in complex backgrounds by utilizing dynamic interpolation and the fusion of multiple features.
- We construct the Multi-scale context reconstruction pyramid network (MCRPN). This network aims to reconstruct spatial features and extract pyramid context, effectively integrating feature information from different levels and enhancing contextual awareness, thereby improving the model’s detection performance.
- We conducted extensive ablation and comparative experiments on the two datasets, and the results show that EggplantDet outperforms other advanced detection algorithms in detection performance, even surpassing the advanced detection model YOLO11.

## 2 Materials and methods

### 2.1 Materials

**Figure 2:**
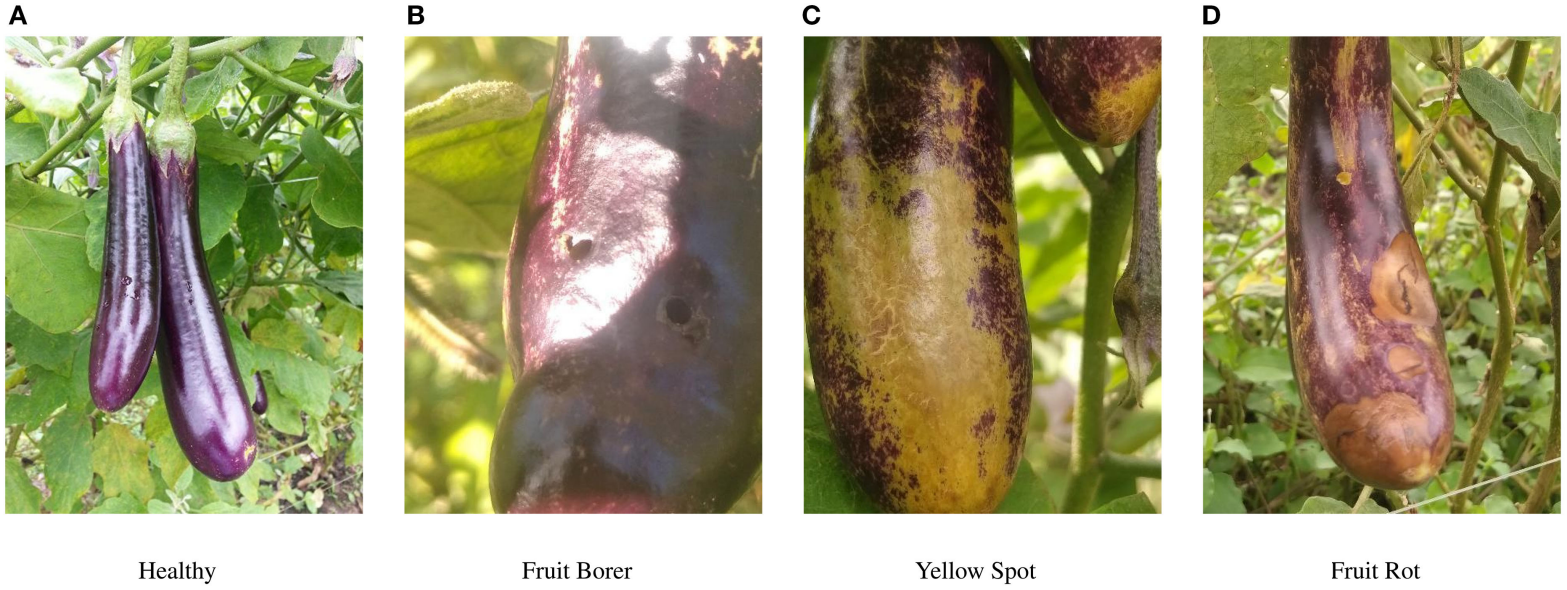
Display of some four eggplant diseases before enhancement. **(A)** Healthy **(B)** Fruit Borer **(C)** Yellow Spot **(D)** Fruit Rot.

Dataset processing: We validated the effectiveness of the proposed model on two datasets: PlantDoc (Singh et al., 2019) and eggplant disease. PlantDoc is a dataset of 2,569 images across 13 plant species and 30 classes (diseased and healthy) for image classification and object detection. There are 8,851 labels. Among them, the eggplant disease data is an eggplant fruit disease dataset from the Roboflow platform, containing four distinct disease categories (BSCS, 2024). The dataset includes four categories: healthy, fruit borer, yellow spot, and fruit rot. Detailed category distributions are presented in **Figure 2**. It is divided into training, validation, and test sets, comprising 2507, 744, and 365 images, respectively. The dataset was collected from diverse, natural cultivation environments, making it highly valuable for applied research.

**Figure 3:**
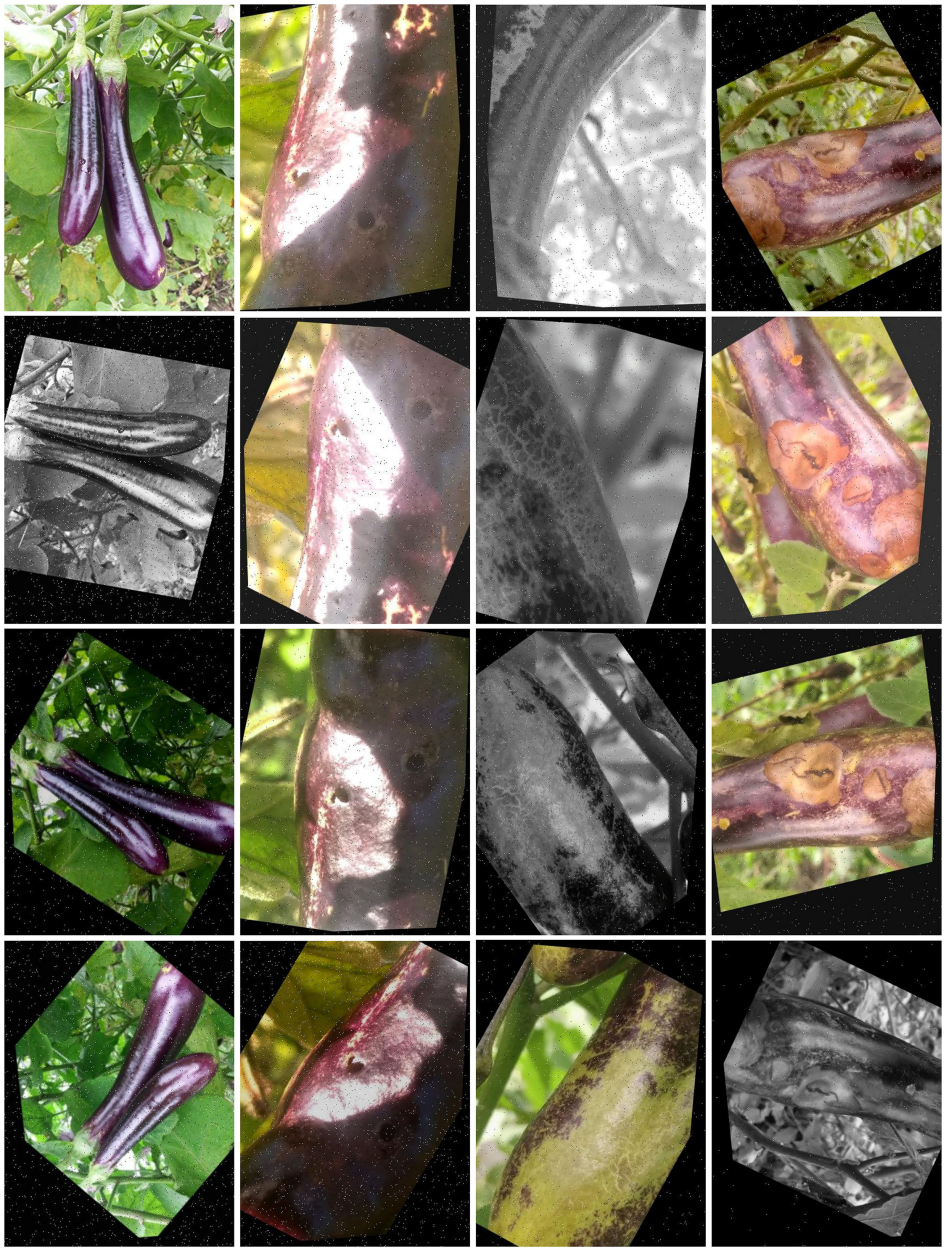
Display of some four eggplant diseases after enhancement.

To better enhance the model’s generalization ability and detection performance on the eggplant disease dataset, we utilized the online data augmentation method of the Roboflow platform to perform data augmentation on the training dataset of this eggplant disease dataset. The augmentations included: 90° rotation (clockwise and counter-clockwise), saturation adjustment (-30% to +30%), general rotation (-45° to +45°), horizontal and vertical flipping, grayscale (applied to 15% of images), hue adjustment (-15° to +15°), cropping (0-20% zoom), brightness adjustment (-15% to +15%), exposure adjustment (-10% to +10%), Gaussian blur (up to 4.8 px), noise addition (up to 1.99% of pixels), and shear transformation (± 15° horizontally and vertically) in **Figure 3**. As a result of these 12 augmentation methods, the expanded dataset includes 7521 images for training, 744 for validation, and 365 for testing.

Implementation details: This study was implemented using a Python deep learning framework on the Windows 11 operating system. For the training process, a batch size of 16 was used, with the SGD optimizer, an initial learning rate of 0.01, weight decay set to 0.0005, and the training was carried out over 100 epochs. The detailed experimental settings are provided in **Table 1**.

**Table 1:** Experimental environment.

Name	Details
Programming language	Python 3.9
GPU	NVIDIA GeForce RTX 4090
CUDA	11.8
Pytorch	2.0.1
Platform	Visual Studio Code

### 2.2 Methods

#### 2.2.1 Macroscopic architecture of EggplantDet

**Figure 4:**
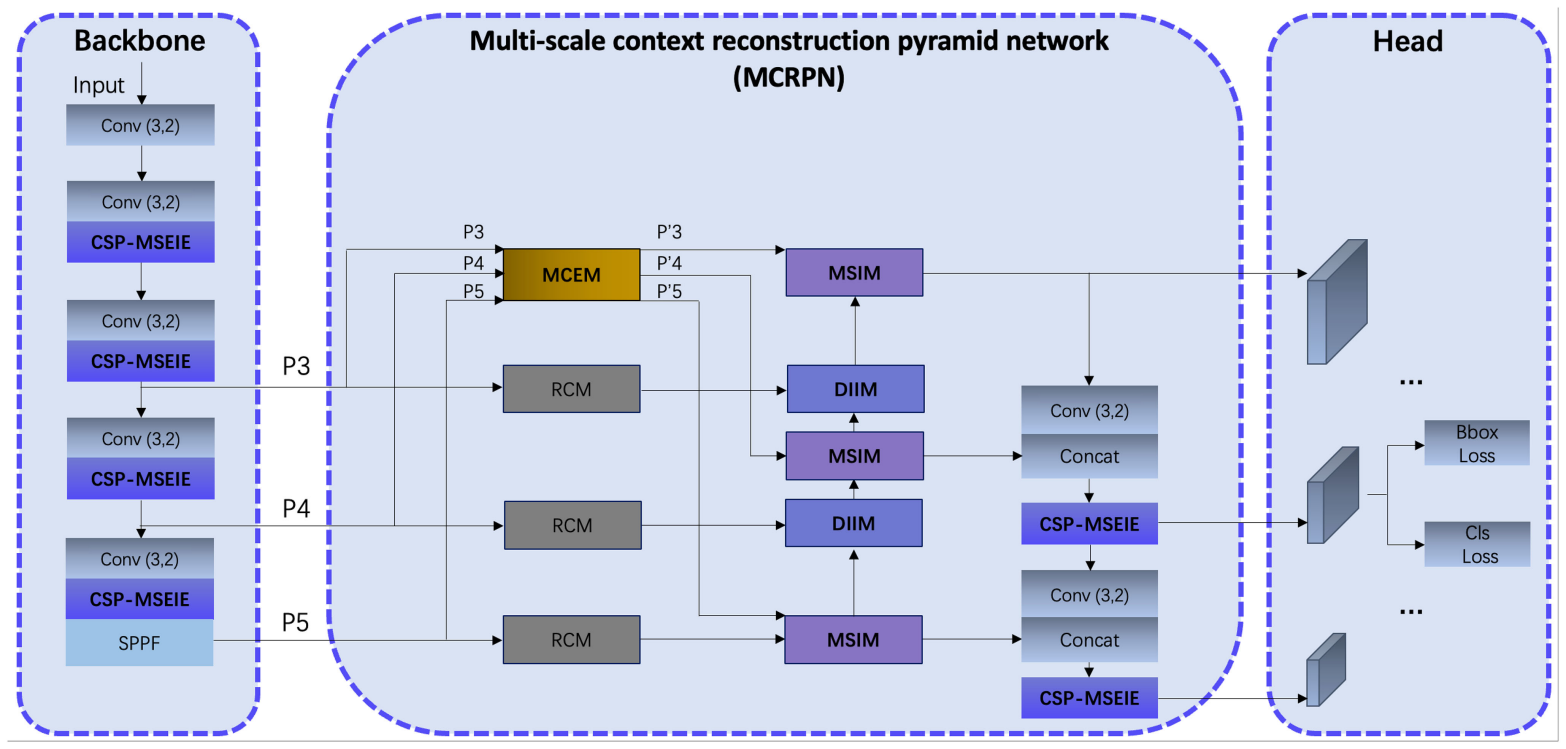
The overall architecture of EggplantDet.

Considering the scale variations of eggplant disease targets and their susceptibility to complex background interference, we constructed EggplantDet based on the YOLOv8 model. **Figure 4** illustrates the overall architecture of the proposed EggplantDet. The detection network comprises three main components: the Backbone, the Multi-scale Context Reconstruction Pyramid Network (MCRPN), and the Head.

- Backbone: The input feature map size is 640×640×3, utilizing multiple 3×3 convolutions to reduce image dimensions and increase channel numbers. To extract features across various disease scales and emphasize the edge information of the diseases, the CSP-MSEIE module was designed and integrated into the Backbone (as shown in **Figure 3**). The convolution, CSP-MSEIE, and SPPF modules work together to generate P3 features of 80×80×256, P4 features of 40×40×512, and P5 features of 20×20×1024 for the subsequent MCRPN network.
- MCRPN: As depicted in the center of **Figure 3**, P3, P4, and P5 are first processed through the RCM (Ni et al., 2024) module to reconstruct and extract key contextual features in both horizontal and vertical directions. Subsequently, the MCEM module integrates features from different levels, while the MSIM and DIIM modules fuse multi-scale features. This significantly improves target recognition performance in complex backgrounds.
- Head: The detection head integrates features from three scale layers: P3, P4, and P5. This design effectively captures fine-grained information in low-level feature maps, thereby enhancing detection accuracy for multi-scale targets. In terms of loss functions, the model retains traditional box and classification losses to ensure accurate prediction box locations and categories.

Overall, the model applies targeted optimizations to both the Backbone and Neck components. Specifically, the introduction of the CSP-MSEIE module and MCRPN network significantly enhances the extraction and fusion of multi-scale and edge features, enabling EggplantDet to exhibit greater robustness and accuracy in eggplant disease detection tasks.

#### 2.2.2 Cross-Stage Partial - Multi-scale Edge Information Enhance (CSP-MSEIE)

**Figure 5:**
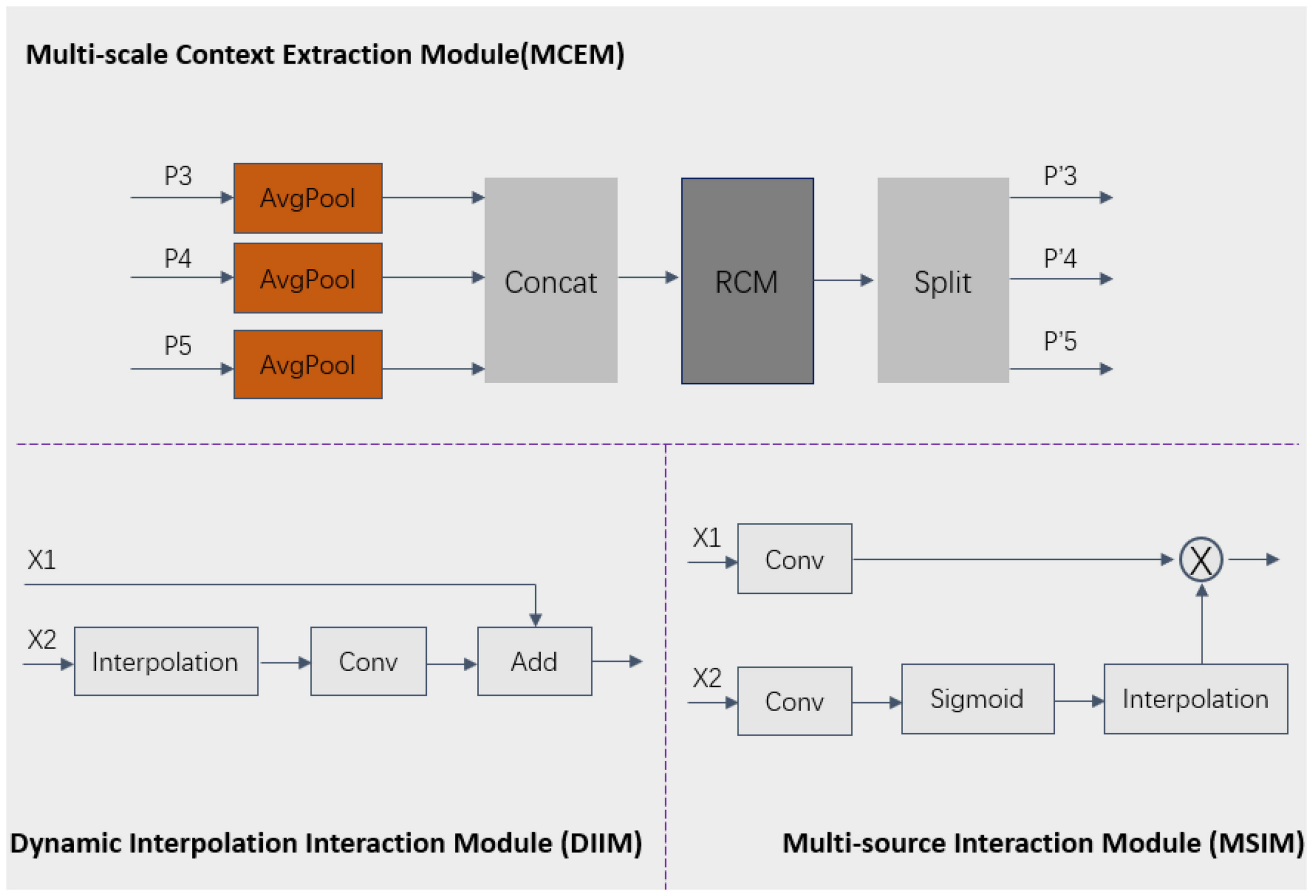
The structure details of the MCEM, MSIM and DIIM.

**Figure 6:**
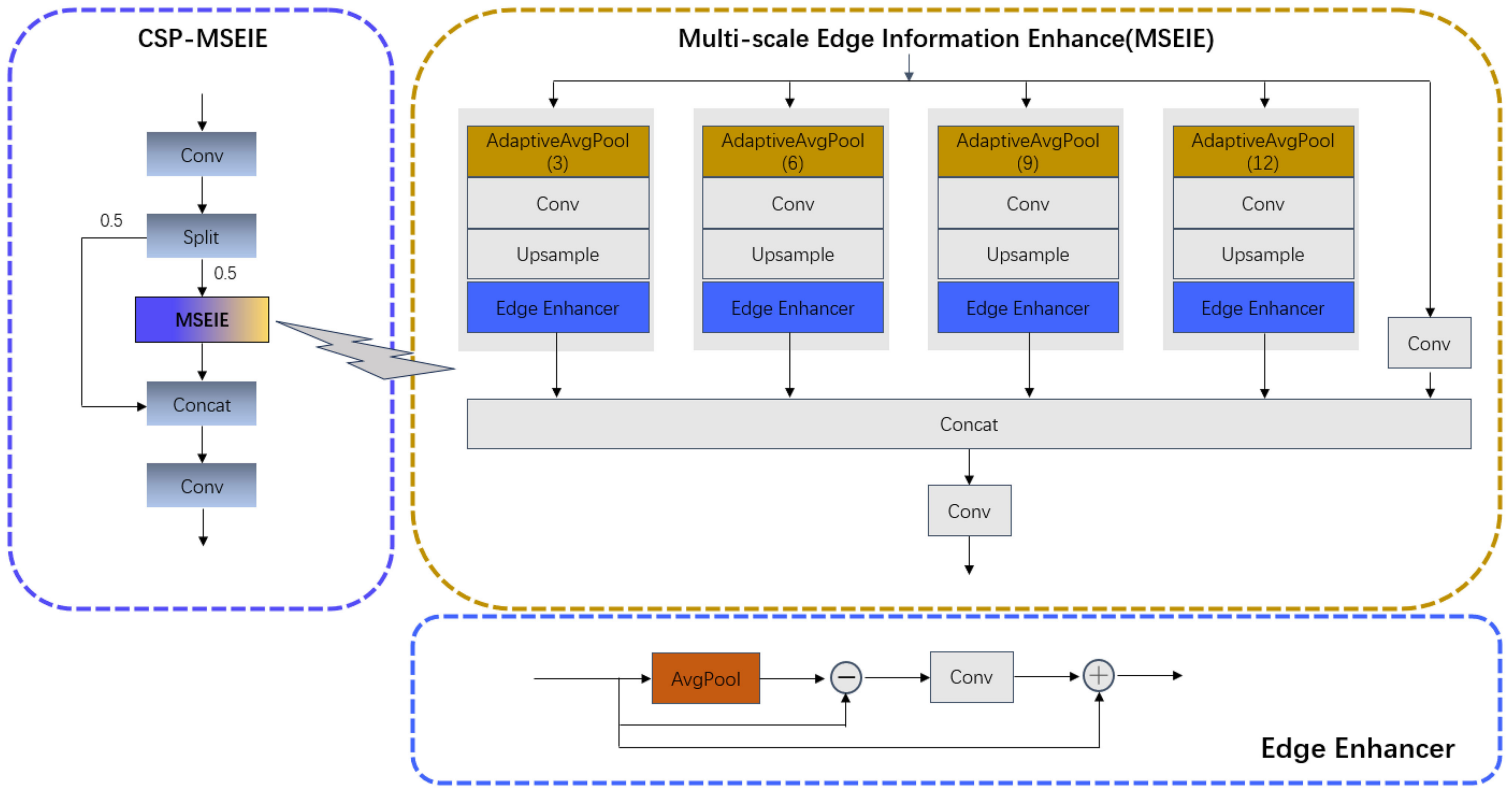
The structure of the CSP-MSEIE.

To extract multi-scale features and emphasize target edge information, we designed the Multi-scale Edge Information Enhance (MSEIE) module. We integrated it with the Cross Stage Partial Net (CSP) structure to form the Cross Stage Partial-Multi-scale Edge Information Enhance (CSP-MSEIE) module, enhancing the learning capability of convolutional neural networks in the Backbone. As depicted in **Figure 5**, the MSEIE module is comprised of three main components: (1) Multi-scale feature extraction: Different parameters of AdaptiveAvgPool (3, 6, 9, 12) are used to achieve multi-scale pooling, extracting local information of different sizes, which helps capture hierarchical features of images. (2) Edge enhancement: The Edge Enhancer module is specifically designed to extract disease edge information, thereby enhancing the network’s sensitivity to edge features. As illustrated in **Figure 6**, the Edge Enhancer module initially applies average pooling to the input feature map to capture low-frequency information. Next, the smoothed feature map is subtracted from the original input feature map to extract the enhanced edge information (high-frequency details). Finally, this high-frequency information is added back to the original feature map to produce the enhanced output. (3) Feature fusion: Features from various scales are aligned to a unified scale through interpolation operations, and after concatenation, they are fused through convolutional layers into a unified feature representation, thus improving the model’s perception of multi-scale features. The CSP-MSEIE module integrates multi-scale feature extraction, edge information enhancement, and convolution operations. Incorporating the CSP-MSEIE module into the Backbone notably enhances edge features and the model’s capacity to extract features.

#### 2.2.3 The principle and details of the MCEM, MSIM and DIIM

To more effectively reconstruct spatial features and capture multi-scale contextual information, we designed three key modules: Multi-scale Context Extraction Module (MCEM), Multi-source Interaction Module (MSIM), and Dynamic Interpolation Interaction Module (DIIM). In the MCEM module, for the P3, P4, and P5 level features extracted by the Backbone, average pooling is first applied to unify feature scales and perform fusion, followed by the use of the RCM module to model axial global context for extracting rectangular key region features. Finally, the features P’3, P’4, and P’5 features are generated through the split operation, thereby effectively integrating information from different levels and enhancing the contextual awareness of the MCRPN network. In the MSIM module, convolution operations are first used to adjust the number of channels, then the sigmoid function and interpolation algorithm further adjust feature dimensions, followed by the multiplication of features from two branches. In the DIIM module, interpolation operations automatically adjust the dimensions of matching features, followed by convolution operations for additive fusion. These three modules greatly enhance the model’s capability to capture features across multiple scales and enhance target recognition performance in complex backgrounds by employing dynamic interpolation and the fusion of multiple features. This process can be specifically expressed in Equations 1–3:


(1) $${\text{output}}_{\text{MCEM}}=\text{Split}\left(\text{RCM}\left(\text{C}\left(\text{AP}\left(P3,P4,P5\right)\right)\right)\right)1$$

(2) $${\text{output}}_{\text{MSIM}}=X_{1}+\left(\text{Interpolation}\left(\text{Conv}\left(X_{2}\right)\right)\right)2$$

(3) $${\text{output}}_{\text{DIIM}}=X_{1}\times \text{Interpolation}\left(S\left(\text{Conv}\left(X_{2}\right)\right)\right)3$$

*Where* AP(·) *represents the average pooling operation*, S *represents the h-sigmoid function*, + *denotes addition operation*, × *denotes multiplication operation*, *and* C(·) *is the Concatenation operation*.

## 3 Experiments

### 3.1 Experimental indicators

In this study, we used several indicators to assess the performance of our model: GFLOPs, Parameters, mean Average Precision (mAP50-90), mean Average Precision (mAP50), and Frames per second (FPS). Of these, mAP50 was selected as the primary evaluation metric. The procedure for calculating the mean Average Precision is described in Equations 4–7.


(4) $$\text{Precision}=\frac{TP}{TP+FP}$$

(5) $$\text{Recall}=\frac{TP}{TP+FN}$$

(6) $$AP=\int_{0}P\left(R\right)\text{ d}R$$

(7) $$\text{mAP}=\frac{\sum_{i=1}^{K}AP_{i}}{K}$$

The variable K signifies the total count of distinct object classifications within the dataset, while each class’s precision is quantified by its specific Average Precision (AP) score. In the performance evaluation equations, several key indicators are utilized: True Positives (TP) represent accurately identified instances of the target condition, False Positives (FP) indicate cases where the algorithm incorrectly flagged non-existent conditions as present, and False Negatives (FN) encompass actual occurrences of the condition that the system failed to recognize.

### 3.2 Comparison studies

To verify the generalization ability of the proposed detection model, this paper first compared the disease detection performance of current mainstream advanced object detection models on the public PlantDoc dataset. As shown in **Table 2**, compared with the baseline model, EggplantDet achieved improvements of 4.3% and 6.7% in mAP50 and mAP50–95, respectively. Compared with the advanced YOLO11n, EggplantDet improved mAP50–95 by 3.0% and mAP50 by 1.6%. Additionally, in terms of Frames per second (FPS), EggplantDet also outperformed other advanced mainstream detection models.

**Table 2:** Comparison with advanced object detection models on the PlantDoc dataset.

Model	Params	GFLOPs	mAP50-95	mAP50	FPS
YOLOv8n (baseline) (Redmon and Farhadi, 2025)	3.0M	8.1	0.285	0.420	190.4
YOLOv9t (Wang et al., 2024b)	2.1M	7.6	0.290	0.423	193.4
YOLOv10n (Wang et al., 2024a)	2.3M	6.6	0.286	0.428	209.5
YOLOv11n (Jocher and Qiu, 2024)	2.6M	6.3	0.295	0.431	227.6
EggplantDet (Ours)	3.1M	7.7	0.304	0.438	241.2

**Figure 7:**
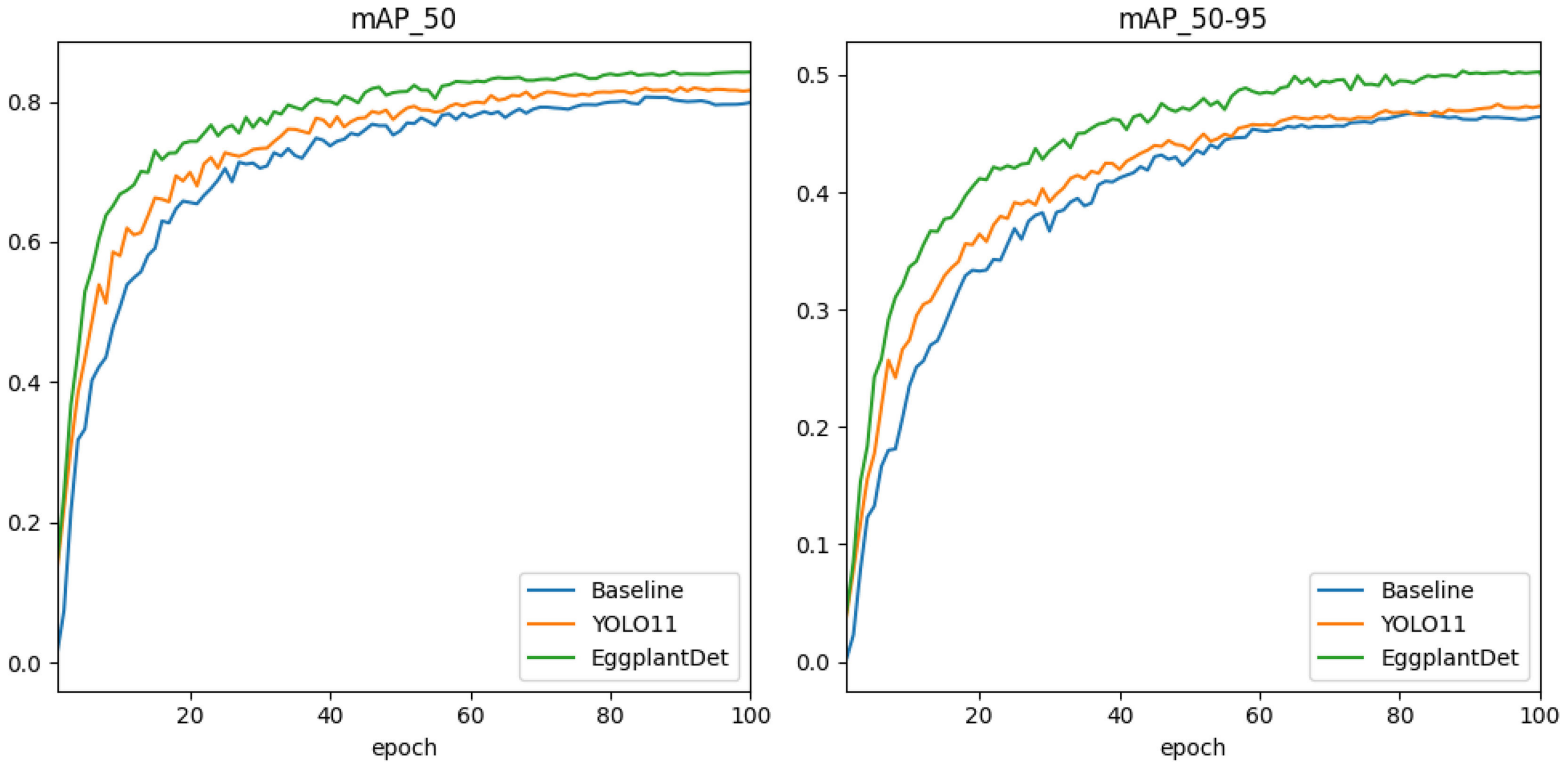
Comparison of detection accuracy during training of different models on the eggplant disease dataset.

Secondly, to further verify the advantages of the proposed eggplant disease detection model, we conducted a comprehensive experimental comparative evaluation on the augmented eggplant disease dataset. **Table 3** presents the experimental results of several advanced detection algorithms, including both two-stage and mainstream single-stage models for comparison. As shown in **Table 3**, two-stage detectors (e.g., SSD and Faster R-CNN) exhibited significantly lower mAP and FPS compared to the proposed method. Additionally, they required substantially more parameters and GFLOPs than the other algorithms. Among single-stage detectors, EggplantDet achieved the best mAP50–95, mAP50, and FPS, while maintaining similar parameter counts and GFLOPs. Compared to the baseline YOLOv8n, EggplantDet achieved 84.3% mAP50 and 50.3% mAP50–95, with respective improvements of 4.7% and 7.2%. Notably, EggplantDet outperformed the state-of-the-art YOLO11n by 0.017 in mAP50, 0.027 in mAP50–90, and 19.7 in FPS. **Figures 7A, B** visually demonstrate the mAP comparison between EggplantDet and the baseline model on the enhanced eggplant dataset, indicating EggplantDet’s excellent performance throughout the process. In conclusion, the improved EggplantDet network demonstrates excellent performance in both detection accuracy and speed, possessing high practical value.

**Table 3:** Detection results with different models on the eggplant disease dataset.

Model	Params	GFLOPs	mAP50-95	mAP50	FPS
Faster-RCNN (Ren et al., 2017)	314M	341.2	0.451	0.721	92.7
SSD (Liu et al., 2016)	53M	112.5	0.418	0.682	44.6
RT-DETR (Zhao et al., 2024)	82M	109.6	0.447	0.728	109.2
YOLOv3 (Redmon and Farhadi, 2018)	12M	19.0	0.423	0.746	80.1
YOLOv5n (Jocher et al., 2022)	2.5M	7.1	0.439	0.763	92.9
YOLOv6 (Li et al., 2022)	4.2M	11.8	0.438	0.755	106.6
YOLOv7 (Wang et al., 2022)	5.6M	13.4	0.426	0.768	118.5
YOLOv8n (Baseline) (Redmon and Farhadi, 2025)	3.0M	8.1	0.469	0.805	186.1
YOLOV8s (Redmon and Farhadi, 2025)	11.1M	28.4	0.478	0.815	108.1
YOLOv9t (Wang et al., 2024b)	2.0M	7.6	0.463	0.810	203.7
YOLOv10n (Wang et al., 2024a)	2.3M	6.5	0.474	0.815	226.1
YOLOv11n (Jocher and Qiu, 2024)	2.6M	6.3	0.476	0.826	250.8
EggplantDet (Ours)	3.1M	7.7	0.503	0.843	270.5

### 3.3 Ablation studies

To evaluate the effectiveness of the proposed modules, YOLOv8 was adopted as the baseline model, and each module was tested individually on the enhanced eggplant disease dataset. The ablation results for the proposed modules are presented in **Table 4**. Initially, the CSP-MSEIE, MCRPN, and MCEM modules were introduced individually. Each module contributed to improvements in detection performance. Specifically, introducing CSP-MSEIE alone yielded the highest improvement in mAP50, whereas MCRPN contributed the most to mAP50–95. Subsequently, the modules were combined in pairs. All three combinations further enhanced detection performance, demonstrating strong synergy among the modules. Notably, the combination of CSP-MSEIE and MCRPN resulted in mAP50–95, mAP50, and FPS increasing to 49.3%, 83.7%, and 266.5, respectively. Finally, all three modules were integrated simultaneously. As shown in the last row of **Table 4**, combining the proposed modules improved the model’s mAP50 and mAP50–95 by 4.7% and 7.2%, respectively, with FPS reaching 270.5. This further confirms the efficacy of the proposed CSP-MSEIE, MCRPN, and MCEM modules in detecting eggplant diseases.

**Table 4:** The results of the ablation study on the eggplant disease dataset.

Baseline	CSP-MSEIE	MCRPN	MCEM	Parameters	GFLOPs	mAP50-95	mAP50	FPS
				3,006,428	8.1	0.469	0.805	186.1
	✓			2,855,900	7.6	0.474	0.823	221.2
		✓		3,339,492	8.3	0.481	0.818	217.7
			✓	3,114,612	8.1	0.467	0.816	209.8
	✓	✓		3,237,964	7.8	0.493	0.837	266.5
	✓		✓	3,034,108	7.9	0.489	0.832	251.4
		✓	✓	3,439,628	8.2	0.487	0.828	247.8
EggplantDet(ours)	✓	✓	✓	3,183,580	7.7	0.503	0.843	270.5

### 3.4 Visual comparative studies

**Figure 8:**
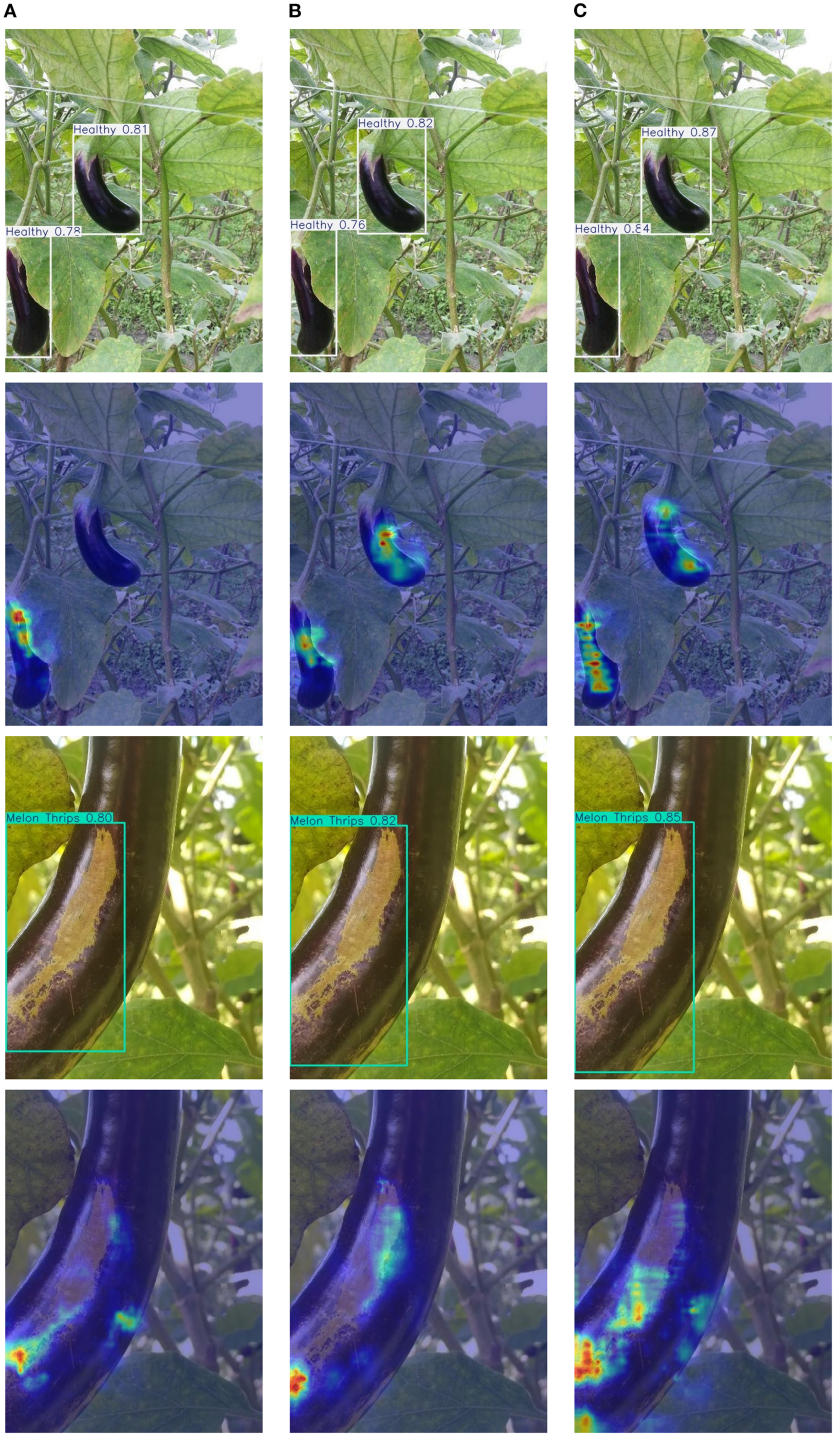
The visualization detection results of different models, **(A)** Baseline Model; **(B)** YOLO11; **(C)** EggplantDet.

**Figure 9:**
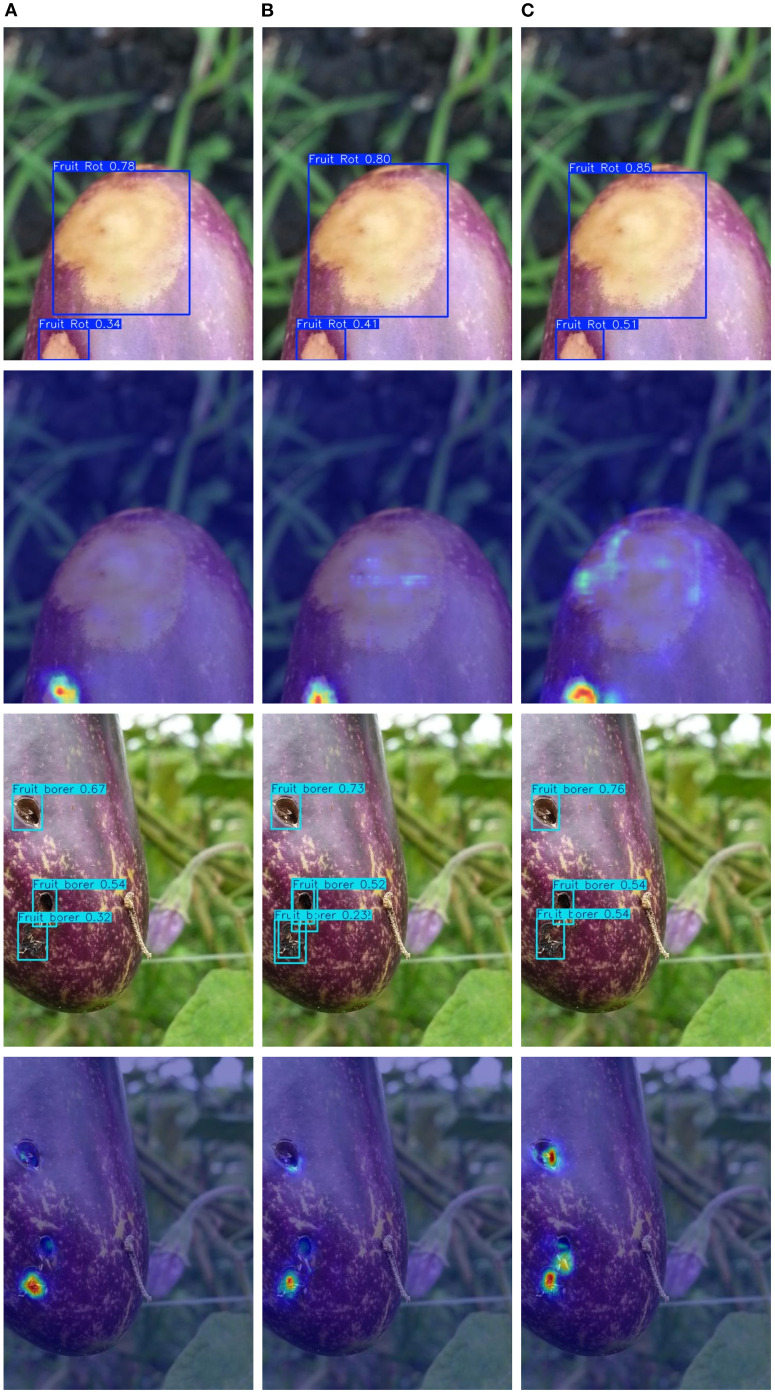
The visualization detection results of different models, **(A)** Baseline Model; **(B)** YOLO11; **(C)** EggplantDet.

**Figures 8**, **9** demonstrate EggplantDet’s detection results compared with those of the baseline model and advanced model on the eggplant disease dataset. The figures visually confirms the proposed detection network’s advantages. The results show that EggplantDet achieves the highest detection accuracy across all four categories, significantly outperforming both the baseline and advanced model YOLO11n. These results suggest that the proposed model outperforms others in eggplant disease detection. Consequently, it offers a promising solution for crop disease detection tasks.

## 4 Conclusion

Crop pest and disease detection technology provides strong support for the development of smart agriculture. To address challenges such as disease scale variations, blurred edge features, and background interference in eggplant diseases, this paper proposes an eggplant disease detection network based on multi-scale edge feature enhancement (EggplantDet), which effectively improves the detection accuracy and localization precision of diseased areas while enhancing detection speed. In the feature extraction stage, the CSP-MSEIE module is incorporated to capture hierarchical features, and the EdgeEnhancer module is used to extract edge information, thereby enhancing the network’s sensitivity to edges. In the feature processing stage, the MCRPN network captures multi-scale contextual information in horizontal and vertical directions and obtains axial global context to explicitly model rectangular key regions, effectively integrating feature information from different levels. Finally, a range of data augmentation techniques is applied to enhance the eggplant disease dataset, thereby boosting the detection model’s ability to generalize. The enhanced detection network outperforms the advanced object detection model YOLO11n, in both detection accuracy and speed. In the future, we will continue to research disease detection networks for more crop varieties and explore lightweight and efficient pest and disease detection technologies to accelerate the transformation of research results into precision crop cultivation applications.

Future work will focus on extending disease detection networks to additional crop species and exploring lightweight, efficient detection technologies to accelerate the deployment of intelligent pest and disease monitoring in precision agriculture.

## Data Availability

Publicly available datasets were analyzed in this study. This data can be found here: https://public.roboflow.com/object-detection/plantdoc/; https://universe.roboflow.com/bohol-island-state-university-vgjlb/eggplant-disease-detection.
